# Laser interstitial thermal therapy enhances bidirectional blood-brain barrier permeability in glioblastoma

**DOI:** 10.1093/neuonc/noag080

**Published:** 2026-04-13

**Authors:** Ryan T Cleary, Yiwei Fu, David Giles, Jinyun Yuan, Diogo P Moniz Garcia, Danny Palmer, Rowland H Han, Timothy Woodiwiss, Alicia B Yang, Dimitrios Mathios, Hong Chen, Albert H Kim

**Affiliations:** Taylor Family Department of Neurosurgery, WashU Medicine, St. Louis, Missouri, USA; Taylor Family Department of Neurosurgery, WashU Medicine, St. Louis, Missouri, USA; The Department of Musculoskeletal Oncology, First Affiliated Hospital of Sun Yat-sen University, Guangzhou, China; Taylor Family Department of Neurosurgery, WashU Medicine, St. Louis, Missouri, USA; Taylor Family Department of Neurosurgery, WashU Medicine, St. Louis, Missouri, USA; Taylor Family Department of Neurosurgery, WashU Medicine, St. Louis, Missouri, USA; University of Denver, Denver, Colorado, USA; Taylor Family Department of Neurosurgery, WashU Medicine, St. Louis, Missouri, USA; Taylor Family Department of Neurosurgery, WashU Medicine, St. Louis, Missouri, USA; Icahn School of Medicine at Mount Sinai, New York, New York, USA; Taylor Family Department of Neurosurgery, WashU Medicine, St. Louis, Missouri, USA; The Brain Tumor Center at Siteman Cancer Center, St. Louis, Missouri, USA; Taylor Family Department of Neurosurgery, WashU Medicine, St. Louis, Missouri, USA; The Brain Tumor Center at Siteman Cancer Center, St. Louis, Missouri, USA; Department of Biomedical Engineering, Washington University in St. Louis, St. Louis, Missouri, USA; Taylor Family Department of Neurosurgery, WashU Medicine, St. Louis, Missouri, USA; The Brain Tumor Center at Siteman Cancer Center, St. Louis, Missouri, USA

**Keywords:** caveolae-mediated transcytosis, ctDNA, glioblastoma, laser interstitial thermal therapy

## Abstract

**Background:**

Laser interstitial thermal therapy (LITT) is a minimally invasive treatment for glioblastoma that increases blood-brain barrier (BBB) permeability. However, the mechanisms and spatiotemporal features of this effect remain unclear. It is also unknown whether LITT promotes release of circulating tumor DNA (ctDNA).

**Methods:**

Using our previously developed LITT mouse model, we employed single-cell RNA-sequencing (scRNA-seq) to investigate gene expression changes in endothelial cells following LITT in the naive mouse brain. Brains were also harvested at multiple time points to assess LITT effects on tight junction (TJ) integrity and transcytosis via immunofluorescence and transmission electron microscopy. Human glioblastoma tissues were analyzed to monitor ambient caveolae-mediated transcytosis in tumor regions. Finally, ctDNA levels in mouse plasma were quantified using digital-droplet PCR following LITT.

**Results:**

LITT triggered downregulation of pathways related to cell-cell junctions and upregulation of transcytosis. LITT transiently disrupted TJ integrity for 7 days up to 100 µm away from the ablation zone. Further, LITT increased caveolae-mediated transcytosis in endothelial cells for 21 days, peaking at 14 days, in part due to suppression of the transcytosis inhibitor Mfsd2a. LITT facilitated ctDNA detection in mouse plasma at 1 h, 3 days, 7 days, and 14 days post-procedure.

**Conclusions:**

Our study demonstrates that LITT transiently disrupts the BBB in the periphery of the ablation with endothelial TJ disruption and transcytosis upregulation following distinct time courses. LITT effects on the BBB are bidirectional and enhance ctDNA release in addition to enabling brain entry of agents.

Key Points

- LITT transiently disrupts tight junctions and elevates transcytosis.
- LITT induces bidirectional BBB permeability and facilitates ctDNA detection.

Importance of the Study

Glioblastoma outcomes remain poor largely due to the blood-brain barrier (BBB), which limits therapeutic delivery. Laser interstitial thermal therapy (LITT) transiently opens the BBB, but the underlying mechanisms and spatiot­emporal dynamics are not fully defined. Our study shows that LITT transcriptionally modulates endothelial cells, transiently alters BBB integrity in a spatially localized manner, and enhances caveolae-mediated transcytosis, facilitating release of tumor DNA into the bloodstream. These findings provide mechanistic insight into LITT-induced BBB modulation and highlight its potential to improve therapeutic access and tumor biomarker detection in GBM.

Glioblastoma (GBM) is the most aggressive and common primary brain cancer in adults with a median survival of approximately 15 months even with aggressive multimodal therapy.^1^^,^^2^ Despite significant advances in our understanding of GBM biology, effective therapies remain limited in part due to the presence of the blood-brain barrier (BBB), which hinders drug delivery to tumor tissues. The limited penetration of the brain imposed by the BBB is primarily governed by 2 cellular mechanisms. One is the specialized tight junction (TJ), which connects brain endothelial cells to restrict paracellular movement of free molecules from the bloodstream to the brain. This structure is composed of junctional proteins, including claudins, occludins, and junction adhesion molecules.^3^ Another process recently found to regulate BBB permeability is endothelial cell transcytosis, which is molecularly less characterized.^4^ Transcytosis allows trans-endothelial cargo transport through different endocytic vesicles, including caveolae.^5^ Caveolae-mediated transcytosis is thought to be the dominant pathway of transcytosis that regulates BBB permeability.^6^ In physiological conditions, brain endothelial cell transcytosis is actively suppressed to limit molecular flow between the bloodstream and the brain parenchyma.^4^ Recent studies identified Mfsd2a (major facilitator superfamily domain-containing 2a) as a key regulator of caveolae-mediated transcytosis in the brain, which is constitutively expressed in brain endothelial cells and inhibits caveolae-mediated transcytosis.^7^^, ^^8^

Intriguingly, laser interstitial thermal therapy (LITT), a minimally invasive surgical technology, has been shown to have BBB-modifying properties in human patients and mouse models.^9^^,^^10^ LITT triggers tumor cell death in a highly controlled manner through thermal ablation,^11^ with patient outcomes comparable to conventional surgical resection in GBM patients in both the newly diagnosed and recurrent settings.^12^ Moreover, LITT induced temporary BBB disruption, occurring within 1-2 weeks after laser ablation and resolving by approximately 4 weeks in GBM patients.^10^ In a mouse model of LITT, we previously showed that LITT disrupts the BBB with kinetics similar to those of patients and, when combined with systemic administration of doxorubicin, a typically brain-impermeable drug, enables brain entry of doxorubicin to improve survival in GBM-bearing mice.^9^

LITT transiently disrupts the BBB through alterations in TJs and transcytosis, potentially enabling bidirectional transport.^13^^,^^14^ Under normal conditions, the restrictive BBB prevents GBM-derived material from entering the circulation, limiting the detection of circulating biomarkers.^15^ By contrast, LITT-induced permeability may enable tumor-derived components—particularly nucleic acids—to escape into the blood. Circulating tumor DNA (ctDNA), small DNA fragments shed by tumor cells, provides a measurable proxy for this process. Thus, enhanced ctDNA detection after LITT could serve as a direct readout of BBB permeability and tumor material efflux. Leveraging LITT to amplify ctDNA signals presents a novel and unexplored approach for monitoring treatment-induced tumor release in GBM.

Previously, we showed that LITT can induce BBB disruption with increased permeability for up to 4 weeks.^9^ However, the mechanistic basis and the spatial and temporal profiles of these effects remain poorly defined. Here, we utilized single-cell RNA-sequencing (scRNA-seq) to identify the transcriptomic changes in mouse brain endothelial cells of the BBB after LITT. Further, we evaluated the effect of LITT on TJ integrity and transcytosis at the BBB in our mouse model. Finally, we investigated whether LITT can enhance the release of ctDNA into the bloodstream.

## Methods

### Laser Interstitial Thermal Therapy

All animal experiments were in accordance with a protocol approved by the Animal Studies Committee at Washington University and compliant with the recommendations of the Guide for the Care and Use of Laboratory Animals (NIH). C57BL/6J mice were used in all experiments. Laser ablation methods have been previously described.^9^ In brief, a stereotactic apparatus was used to target coordinates 1 mm rostral and 2 mm lateral to the bregma, and 2 mm deep. The laser was manually controlled to maintain the desired temperature range at 43 °C for 3 min. Sham controls involved creating a burr hole without probe insertion unless specified.

### Tumor Implantation and LITT

For ctDNA-related experiments, 2000 SB28 syngeneic tumor cells, engineered to overexpress eGFP and luciferase, were injected into the mouse brain at coordinates 1 mm rostral and 2 mm lateral to bregma, and 2 mm deep. Sham controls included probe insertion without laser activation. Bioluminescence imaging was employed to confirm and monitor tumor growth. Mice received intraperitoneal (IP) injections of 200 µL of d-Luciferin (15 mg/µL in phosphate-buffered saline [PBS]; Goldbio, #LUCK-1G) and were anesthetized with 2% isoflurane during imaging. Bioluminescence was recorded using the Lago imaging system (Spectral Instruments Imaging). Seven days after the tumor was implanted, laser ablation was performed at coordinates 1 mm rostral and 2 mm lateral to the bregma and switched on and off to maintain the desired temperature at 44⁰C for 90 s at both 2.5 and 0.75 mm deep.

### Brain Endothelial Cell Isolation

Brains were harvested from mice 7 days after treatment with bilateral laser ablation or sham surgery. Evans Blue (Sigma-Aldrich, E2129) was injected into the retro-orbital vein of 7-8-week-old female mice. After 30 min, the mice were anesthetized with isoflurane and perfused with PBS. Brains were harvested, and the tissue stained with Evans Blue was imaged on a fluorescence microscope using the Cy5 channel and then microdissected. A similar volume of tissue was microdissected from sham mice. Brain tissue from 6 mice was pooled and enzymatically dissociated using the Neural Dissociation Kit according to manufacturer instructions (Miltenyi, 130-092-628). Suspensions were filtered through a 70-µm strainer, and myelin was removed by centrifugation in Percoll. The remaining myelin-depleted cell suspension was blocked for 5 min with Fc preblock (CD16/CD32, BD 553141) on ice, then stained for 30 min with anti-CD31-APC (1:100, BD 551262), anti-CD45-FITC (1:100, BD 553080), and anti-CD11b-BV421 (1:100, Biolegend Clone M1/70, 101236) in FACS buffer (0.5%-1% BSA, 2 mM EDTA in PBS). Dead cells were excluded by staining with Sytox orange (1:1000, Thermo Fisher, S34861). Flow cytometry data and cell sorting were acquired on a MoFlo (Beckman Coulter) with FACSDiva software (BD Biosciences). FlowJo software was used for further analysis and depiction of the gating strategy. Gates are indicated by framed areas. Cells were gated on forward (FSC = size) and sideward scatter (SSC = internal structure). FSC-A and FSC-W gating were used to discriminate single cells from cell doublets/aggregates. CD11b^+^ and CD45^+^ cells were gated to exclude monocytes/macrophages and microglia. CD31^+^Cd11b^-^CD45^-^ brain endothelial cells were sorted directly into lysis buffer in 96-well plates (Bio-rad), containing RNase inhibitor (Takara Bio), oligo(dT), dNTPs, and ERCC spike-ins and stored at −80°C for further processing.

### Single-Cell RNA-Sequencing

Cell lysis, first-strand, and cDNA synthesis were performed using the Smart-seq-2 protocol in 96-well formats, as previously described.^16^ After cDNA amplification (23 cycles), cDNA concentrations were measured with a dye-fluorescence assay (Quant-iT dsDNA High Sensitivity kit; Thermo Fisher) on a SpectraMax i3x microplate reader (Molecular Devices). Sample plates were selected for downstream processing if the mean concentration of blanks (ERCC-containing, non-cell wells) was >0 ng/µL and, after linear regression of the values obtained from the Quant-iT dsDNA standard curve, the *R*^2^ value was >0.98. Sample wells were then selected if their cDNA concentrations were at least 1 SD greater than the mean concentration of the blanks. These wells were reformatted to a new 384-well plate at a concentration of 0.3 ng/µL and a final volume of 0.4 µL using an Echo 550 acoustic liquid dispenser (Labcyte). Libraries were prepared and pooled using the Illumina Nextera XT Library Sample Preparation kit. Libraries were then sequenced on the NovaSeq 6000 Sequencing System (Illumina) using 2 × 100 bp paired-end reads and 2 × 8 bp index reads with a 200-cycle kit (Illumina, 20012861). Samples were sequenced at an average of 7.5M reads per cell.

### SMART-Seq-2 Data Analysis

Transcripts run through the SMART-seq-2 protocol were aligned to the mm39 transcriptome with Salmon,^17^ and a gene-level count matrix from TPM-scaled transcript abundance data was created within R using the tximport package.^18^ Subsequent analysis was performed in R with the Seurat package.^19^ Genes were filtered for those with expression in at least 1% of cells. Cells were filtered for those with >500 genes, >75k transcripts, and <10% mitochondrial or ribosomal transcripts. Differentially expressed genes between the Sham and LITT conditions were identified using the FindMarkers function within Seurat (MAST test) and visualized using the EnhancedVolcano package.^20^ Gene set enrichment was determined and visualized using the ClusterProfiler package.^21^ Gene sets of cell junctions and transcytosis-related pathways were acquired by the AnnotationDbi package,^22^ and then pathway scores were compared between the Sham and LITT conditions using the AddModuleScore function within the Seurat package. Violin plots of genes were visualized using the Seurat package.

### Magnetic Resonance Imaging

Mouse brain MRI data were acquired on a Bruker 9.4-T (400-MHz), 20-cm clear bore MRI scanner with a mouse brain CryoProbe and using ParaVision 360 V3.3 software (Bruker, Billerica, MA). Animals were anesthetized with 1.0-%-1.5% isoflurane and injected with 0.1-0.2 mmol/kg of Gadavist (Bayer) contrast agent prior to imaging. T2-weighted (repetition time [TR] = 3000 ms, echo time [TE] = 60 ms) and T1-weighted (TR = 200 ms, TE = 3 ms) images were acquired with a field-of-view of 12.8 × 12.8 mm^2^, a data matrix of 128 × 128, and 9 slices at 1-mm thickness. MRIs were obtained 2 days after LITT to confirm ablation prior to sacrificing animals for harvesting histological specimens.

### Human Tissue Collection

Patient samples used for transmission electron microscopy analyses were derived from patients treated at Barnes-Jewish Hospital (St. Louis, MO) during open tumor resections. All patients provided written informed consent to participate in the study following Institutional Review Board approval (IRB# 201409046, 201111001). Patient samples were collected from either contrast-enhancing or nonenhancing tumor regions or normal brain outside of the tumor volume (based on preoperative MRI and intraoperative neuronavigation). Patient samples were immediately rinsed in cold PBS to remove residual blood, then fixed by drop immersion in 5% glutaraldehyde/4% PFA/0.1M sodium cacodylate for 1 h at room temperature, then overnight at 4 °C as further detailed below.

### Transmission Electron Microscopy

Evans Blue was injected into the retro-orbital vein of 7-8-week-old female C57BL6J mice. After 30 min, the brains were harvested, and the hindbrain and contralateral hemisphere were removed. Brains were fixed by drop immersion, first in 5% glutaraldehyde/4% PFA/0.1M sodium cacodylate for 1 h at room temperature, then overnight at 4 °C in 4% PFA/0.1M sodium cacodylate. Following fixation, brains were washed overnight in 0.1M sodium cacodylate. Coronal vibratome free-floating sections of 50 µm were collected, and Evans Blue staining was used to identify sections through the laser ablation penumbra. Sections were post-fixed in 1% osmium tetroxide and 1.5% potassium ferrocyanide, dehydrated, and embedded in epoxy resin. Ultrathin sections (80 nm) were then cut from the block surface, collected on copper grids, and counterstained with Reynold’s lead citrate. A 1200EX electron microscope (JOEL) equipped with a 2k CCD digital camera (AMT) was used for all TEM studies. Images were analyzed using ImageJ (NIH). Endothelial cells located within 100 µm of the coagulum edge were included for luminal vesicle quantification.

### Immunofluorescence

For claudin-5 and CD31 co-staining, mice were anesthetized with isoflurane and perfused with PBS. Brains were harvested and quickly frozen in OCT (Sigma-Aldrich) using isopentane on dry ice. For Mfsd2a and CD31 co-staining, mice were anesthetized with isoflurane and perfused with PBS and 4% PFA. Brains were harvested, fixed in 4% PFA overnight, cryopreserved in increasing concentrations of sucrose solution (10%, 20%, 30%), and mounted in OCT (Sigma-Aldrich). OCT blocks were stored at −20 °C for 24 h before slicing on a cryostat at 10-µm thickness. Slices were thawed, mounted onto Superfrost Plus (Fisher Scientific) slides, and then stored at −80 °C. Slides used for claudin-5 and CD31 co-staining were post-fixed in ice-cold methanol for 10 min, then briefly washed in PBS. Slides used for Mfsd2a and CD31 co-staining were rehydrated in PBS for 5 min. All slides were then blocked in the blocking buffer containing 1× PBS, 10% goat serum, and 0.2% Triton X-100 at room temperature for 1 h, then incubated at 4 °C overnight with the following primary antibodies: CD31 (1:100, BD Pharminogen 550274), claudin-5 (1:300, Thermo Fisher Scientific 34-1600), and Mfsd2a (1:200, a generous gift from Dr. Chenghua Gu, Harvard Medical School, Boston, MA), followed by incubation with corresponding Alexa fluorophore-conjugated secondary antibodies (1:500, Thermo Fisher Scientific). Fluorescent images were taken using an automated inverted microscope (Leica Microsystems). Analysis of immunofluorescence images was performed via corrected total fluorescence on Nikon Elements.

### Blood Collection and Cell-Free DNA Extraction

Blood was collected from the submandibular vein of mice at different timepoints, 1 h, 3 days, 7 days, and 14 days after LITT/sham surgery. Between 50 and 100 µL of blood was collected into EDTA-coated tubes to prevent hemolysis. Blood samples were centrifuged at 2500 × *g* for 10 min at 4 °C. Then the separated plasma portion was transferred to 1.5-mL tubes and centrifuged at 16,100 × *g* for 10 min at 4 °C to remove additional debris. The plasma/serum RNA/DNA Purification Mini Kit (Norgen Biotek) was used to extract cfDNA from mouse plasma per the manufacturer’s protocol. PBS was added to the plasma samples to make up the volume of 200 µL. cfDNA was eluted in 30 µlLof the corresponding buffer. cfDNA was preamplified using Q5 hot start high-fidelity master mix (New England Biolabs) with forward and reverse primer pairs for enhanced green fluorescent protein (*eGFP*) (same primers used for ctDNA analysis). Pre-amplification was performed using the Eppendorf Mastercycler: 98 °C for 5 min; 12 cycles of 98 °C for 30 s, 60 °C for 1 min; a final extension at 72 °C for 5 min; and 1 cycle at 4°C for infinite time. Preamplified products were directly used for further ddPCR reactions.

### ctDNA Analysis with ddPCR

Custom sequence-specific primers and fluorescent probes were designed and synthesized for *eGFP* detection (Millipore Sigma). The forward and reverse primer sequences for *eGFP* are 5′-GACCACTACCAGCAGAACACC-3′ and 5′-CCAGCAGGACCATGTGATCG-3′, respectively. The *eGFP* probe sequence is 5′-CCGACAACCACTACCTGAGCACCCAGTC-3′, the 6-carboxyfluorescein (6-FAM), and the Black Hole Quencher 1 (BHQ1).ddPCR reactions were conducted using Bio-Rad Q200X according to the manufacturer’s instructions (Bio-Rad). ddPCR reactions were prepared in a 20-µL system with 2× ddPCR Supermix for probes (no dUTP) (Bio-Rad), 5 µL of target DNA product, 0.1 µM forward and reverse primers, and 0.1 µM probes. The QX200 manual droplet generator (Bio-Rad) was used to generate droplets (20.5 µL reaction + 70 µL oil for probes). The PCR step was performed on a C1000 Touch Thermal Cycler (Bio-Rad) by use of the following program: 1 cycle at 95°C for 10 min, 48 cycles at 95°C for 30 s and 60°C for 1 min, 1 cycle at 98°C for 10 min, and 1 cycle at 12°C for 30 min, 1 cycle at 4°C infinite, all at a ramp rate of 2°C/s.

Data were acquired on the QX200 droplet reader (Bio-Rad) and analyzed using QuantaSoft Analysis Pro (Bio-Rad). All results were manually reviewed for false positives and background noise droplets based on negative and positive control samples. Assays were considered positive if >3 droplets exceeded the threshold fluorescence ctDNA concentrations (copies/µL plasma), which were calculated by multiplying the concentration (provided by QuantaSoft) by elution volume, divided by the input plasma volume used during DNA extraction.

### Statistics

Statistical analyses were performed using GraphPad Prism version 10.0 and R version 4.4.2. Pathway scores were compared between conditions using the Wilcoxon rank-sum test, with the *P*-value adjusted for multiple biologically related pathway-level comparisons using the Benjamini-Hochberg false discovery rate method. For experiments comparing 2 groups, unpaired Student’s *t*-tests were used. Comparisons involving multiple timepoints and/or groups were analyzed by 2-way ANOVA with Dunnett’s/Šidák’s multiple comparisons post hoc test. Comparison of ctDNA-positive rates was performed using a two-sided Fisher’s exact test. Data are presented as individual values with mean ± SEM unless otherwise stated, and sample sizes (*n*) are reported in the figures or figure legends. Differences were considered statistically significant at *P* < .05. Statistically significant differences between conditions are indicated by asterisks as follows: ns, *P* > .05; *P < .05; **P < .01; ***P < .001; ****P < .0001.

## Results

### Single-Cell RNA-Seq Analysis of Brain Endothelial Cells Identifies Alterations in Pathways Related to TJs and Transcytosis Following LITT

**Figure 1.**
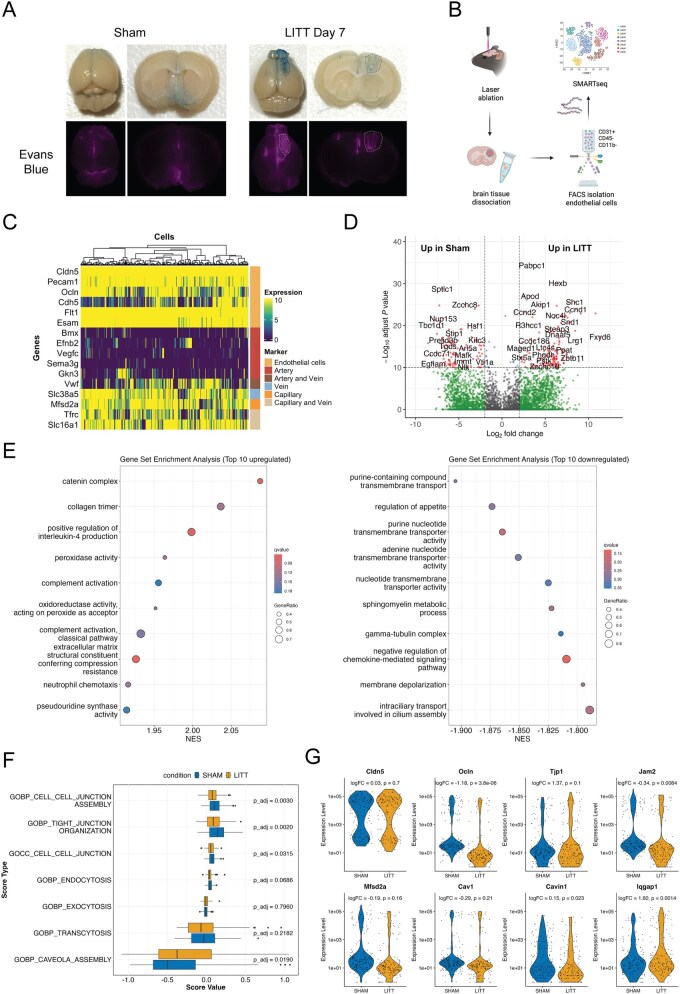
Single-cell RNA-sequencing analysis identified changes in pathways related to tight junctions and transcytosis after laser interstitial thermal therapy (LITT). (A) Representative images show Evans Blue staining in naive brains 7 days after treatment. Dashed lines demarcate the ablation core. (B) Schematic overview of the scRNA-seq experimental pipeline. (C) Expression heatmap of selective endothelial cell markers. (D) Volcano plot depicting genes differentially expressed after LITT (sham as control). (E) Gene set enrichment analysis (GSEA) showing the top 10 upregulated and downregulated Gene Ontology (GO) terms ranked by *q*-value. (F) Geneset scores of cell junctions and transcytosis GO terms of LITT and sham. (G) Violin plots illustrating expression changes in tight junctions and transcytosis-related genes. Abbreviation: NES, normalized enrichment score.

scRNA-seq was performed to investigate transcriptomic changes in brain endothelial cells induced by LITT. Brains were harvested 7 days post-treatment, and tissues were stained with Evans Blue to visualize the vasculature and microdissection. LITT-treated naive brains exhibited increased Evans Blue staining compared with sham controls, reflecting enhanced vascular permeability (Figure 1A). Interestingly, in SB28-bearing brains, Evans Blue staining was also higher after LITT compared with sham, although sham tumors displayed baseline Evans Blue signal, consistent with the inherently disrupted BBB in tumors. Tumor GFP signal was reduced in LITT-treated brains due to tumor ablation (Figure S1). FACS-sorted CD31⁺CD11b⁻CD45⁻ endothelial cells were then subjected to scRNA-seq using the Smart-seq2 protocol^16^ from LITT-treated and sham control samples (Figure 1B). Among CD45⁻CD11b⁻ single cells, CD31⁺ endothelial cells comprised 4.66% (356/7647) in the LITT group and 12.1% (378/3117) in the sham group. After removing lowly expressed genes and low-quality cells, we retained 374 high-quality cells and 10,886 genes for downstream transcriptional analysis. Analysis of marker gene expression confirmed the endothelial identity of these cells, with high expression of Cldn5, Pecam1, Flt1, and Esam observed across the population (Figure 1C). Examination of zonation-specific markers showed low expression of arterial markers (*Bmx, Efnb2, Vegfc, Sema3g, Gkn3*); moderate expression of arterial and venous marker (*Vwf*); and high expression of venous marker (*Slc38a5*), capillary marker (*Mfsd2a*), and capillary and venous markers (*Trfc, Slc16a1*) in the cells. These findings collectively suggest that the profiled cells are endothelial and predominantly consist of capillary and venous subpopulations.

Differentially expressed gene analysis was conducted to identify genes altered by LITT ablation (Figure 1D). Gene Set Enrichment Analysis (GSEA) using Gene Ontology (GO) terms revealed significantly enriched pathways. Notably, the catenin complex pathway was upregulated (Figure 1E); the Wnt/β-Catenin signaling pathway is known to play a crucial role in the regulation of TJ complex assembly in BBB ­endothelial cells.^22^ Additionally, complement activation and neutrophil chemotaxis pathways were upregulated, with downregulation of the negative regulation of chemokine-mediated signaling following LITT, suggesting initiation of an inflammatory/immune response subsequent to laser ablation. Several pathways of nucleotide transmembrane transporter activity were downregulated, suggesting LITT modulates transmembrane transport in endothelial cells.

Utilizing GO terms, geneset scores specifically related to BBB permeability mechanisms were compared between LITT and sham conditions (Figure 1F). The cell-cell junction scores were significantly lower in the LITT group than in the sham group, especially the score related to TJs. In addition, the caveola assembly score was significantly higher in the LITT group, suggesting a higher activity of caveolae-mediated transcytosis. Consistently, violin plots showed significant downregulation of the TJ-related genes *Ocln* and *Jam2* and upregulation of *Cavin1*, a gene that stabilizes caveolae,^23^ and *Iqgap1*, a gene implicated in transcytosis in endothelial cells post-LITT (Figure 1G).^23^

### LITT Induces Localized and Transient TJ Disruption

**Figure 2.**
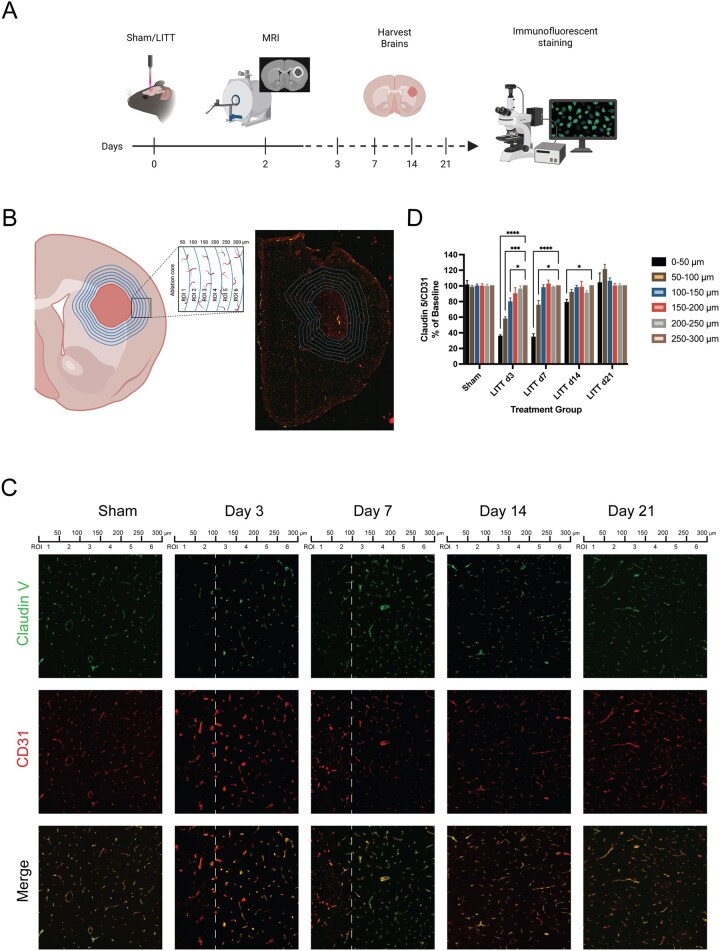
Laser interstitial thermal therapy (LITT) induces a transient decrease in tight junction integrity for up to 7 days and 100 µm away from the ablated core. (A) Schematic illustrating study design with LITT applied at day 0, confirmed with the use of MRI at day 2, and brains harvested at days 3, 7, 14, and 21. (B) Schematic illustration and representative image demonstrating selection of regions of interest (ROIs) in 50-µm increments away from the ablation core for further sectioning and study. (C) Confocal images from immunofluorescence of both sham and LITT-treated brains at different time points, staining for tight junction marker claudin-5 and endothelial membrane protein CD31 Overlay images from both channels were shown (Merge). Dashed vertical lines demarcate the ROIs (1-2), corresponding to 0-100 µm from the ablation core. (D) Quantification of normalized claudin-5 to CD31 ratio of LITT-treated brains showing endothelial tight junction density compared to the sham control at the indicated time points and regions (*n* = 3 for each condition). *P*-value calculated by 2-way ANOVA with Dunnett’s multiple comparisons testing.

To validate the changes observed in the scRNA-seq analysis of LITT-treated brain endothelial cells, we used our LITT mouse model to assess the spatiotemporal impact of LITT on the cellular processes that define BBB permeability.^9^ Laser ablation (vs. sham surgery) was stereotactically delivered into the mouse brain and confirmed 2 days after treatment using MRI (Figure 2A). We first evaluated the effect of LITT on the integrity of brain endothelial TJs. Following LITT, brains were harvested at different time points (post-LITT days 3, 7, 14, and 21), and then brain sections were processed for immunofluorescence using antibodies against the TJ marker claudin-5 and the endothelial cell receptor CD31. Fluorescence intensity was quantified in different regions of interest in 50-µm increments away from the ablation core (Figure 2B). After laser ablation, claudin-5 expression was markedly reduced compared to sham at day 3, and this effect was spatially restricted to an area within 100 µm from the laser centroid (Figure 2C). Claudin-5 downregulation persisted from day 3 through 7, with partial recovery by day 14 and complete recovery by day 21 (Figure 2C and D). Collectively, these results demonstrate that LITT promotes an early and transient decrease in TJ integrity lasting up to 14 days and up to 100 µm away from the ablation region.

### LITT Increases Caveolae-Mediated Transcytosis and Is Associated With Downregulation of Mfsd2a

**Figure 3.**
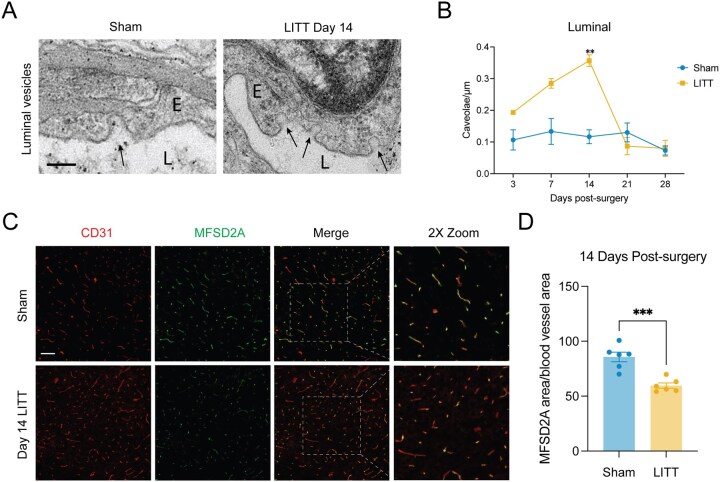
Laser interstitial thermal therapy (LITT) induces a temporary increase in transcytosis for up to 14 days in part due to downregulation of Mfsd2a. (A) Luminal vesicles in endothelial cells (E) facing the luminal area (L) in brain tissue with indicated treatments were visualized by TEM. Caveolae invaginations were indicated. (B) Quantification of luminal vesicles in both LITT-treated brains and sham was shown (n = 3 for each condition, 10 capillary cross-sections per animal). **, *P* < 0.01 by 2-way ANOVA with Dunnett’s multiple comparisons testing. Data are presented as mean ± SEM. (C) Confocal images from immunofluorescence of both sham and LITT-treated brains staining for endothelial membrane protein CD31 and Mfsd2a. Overlay images from both channels were shown (Merge). (D) Quantification of normalized Mfsd2a to blood vessel area of LITT-treated brains and the sham control at day 14 after treatment (*n* = 6 for each condition). **P < 0.01 by Student’s *t*-test. Scale bar: 100 nm (A) and 100 µm (C).

We then assessed the temporal effect of LITT on caveolae-mediated transcytosis. LITT-treated brain slices along with sham surgical controls were collected at different time points up to 4 weeks, and characteristic invaginations of caveolae from the luminal plasma membrane of endothelial cells were visualized by transmission electron microscopy. TEM revealed an increased number of plasma membrane invaginations and caveolae-like vesicular structures in endothelial cells after LITT (Figure 3A). The increased abundance of such structures therefore provides morphological evidence for enhanced endothelial transcytosis following LITT treatment. Compared to sham surgery, LITT ablation resulted in an increase in endothelial cell transcytosis, which peaked at post-LITT day 14 and decreased to control levels by post-LITT day 21 (Figure 3B). These data raised the hypothesis that LITT may disrupt the normal brain suppression of endothelial cell transcytosis, a process mediated by the endothelial cell gene *Mfsd2a.*^7^^,^^8^ In our earlier endothelial cell scRNA-seq data, we observed a trend toward a decrease in *Mfsd2a* after LITT (Figure 1G). We therefore asked whether LITT might induce caveolae-mediated transcytosis through Mfsd2a downregulation in brain endothelial cells by performing Mfsd2a immunofluorescence in laser-treated brains. Indeed, protein expression of endothelial Mfsd2a in LITT-treated brain tissue was downregulated at day 14, the peak time of LITT-triggered endothelial transcytosis, compared to sham-treated brains by immunofluorescence (Figure 3C and D). These results support the hypothesis that Mfsd2a controls not only baseline transcytosis but may also contribute to BBB permeability changes in the context of LITT.

### Baseline Caveolae Vesicular Trafficking in Human GBM

The presence of suppressed baseline endothelial transcytosis in healthy murine models and its disruption in several human disease processes have been previously demonstrated.^23^ However, the basal rate of transcytosis in GBM and its possible variation across tumor regions are unknown. Direct evaluation of human tumor tissue after laser ablation would also be interesting to investigate, but as this would require an otherwise unindicated second tissue biopsy after LITT, obtaining this tissue is not ethically feasible. We thus evaluated the extent of baseline transcytosis in untreated human GBM specimens obtained during open tumor resections. To address this question, we monitored samples from both enhancing and nonenhancing GBM tumor regions as well as normal brains from human patients and subjected them to TEM (Figure S2A). As expected, we observed that normal brain exhibited a very low density of endothelial caveolae, indicating a low ambient rate of endothelial transcytosis. Similarly, nonenhancing tumor regions had low endothelial caveolae density. Interestingly, an almost 2-fold increase in endothelial caveolae density was observed in enhancing tumor regions compared to both nonenhancing regions and normal brain, suggesting a mildly elevated level of endothelial transcytosis in enhancing tumor regions (Figure S2B). Overall, these human data indicate that ambient endothelial transcytosis is extremely low in normal brain and nonenhancing GBM tumor regions with only a slight elevation in enhancing tumor regions.

### LITT Enhances BBB Permeability to Facilitates ctDNA Release

**Figure 4.**
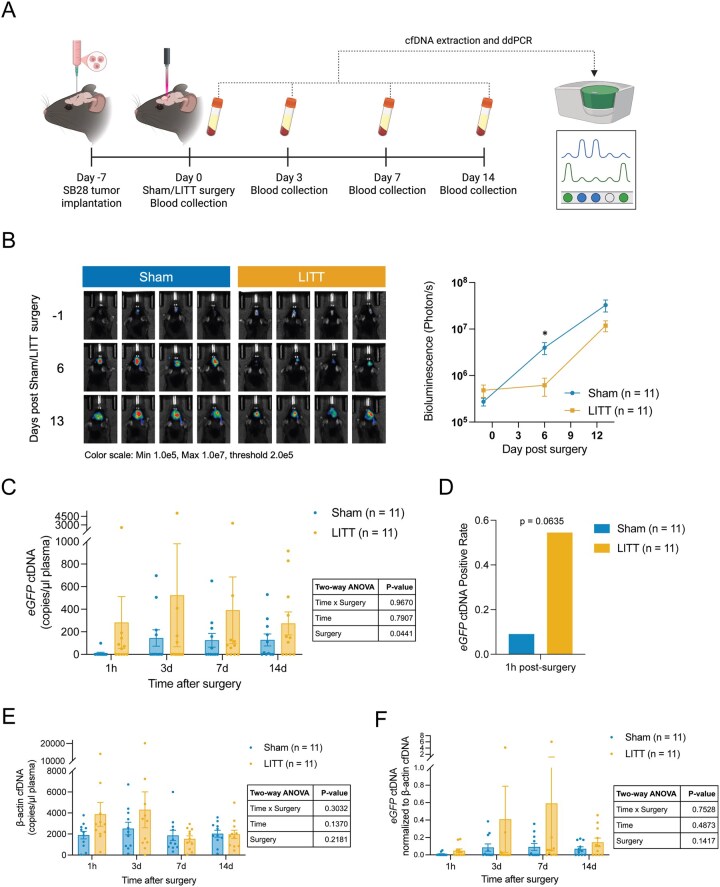
Laser interstitial thermal therapy (LITT) enhances blood-brain barrier (BBB) permeability and facilitates increased ctDNA release. (A) Schematic timeline showing SB28 glioma implantation, LITT or sham surgery, and blood collection time points for ctDNA analysis via ddPCR. (B) Tumor volume measurements showed smaller tumors in the LITT group compared to sham on day 6; no significant differences were observed on day −1 and day 13. *P < .05 by 2-way ANOVA with Šidák’s multiple comparisons testing. (C) Quantification of *eGFP* ctDNA levels in mouse plasma at multiple time points post-surgery. *eGFP* ctDNA was significantly higher in the LITT group compared to sham controls (*P* < .05, 2-way ANOVA). (D) The positive *eGFP* ctDNA detection rate (>5 copies/µL plasma) in the sham and LITT group at 1 h post-surgery (*P* = .0635 by 2-sided Fisher’s exact test). (E) Quantification of β-actin cfDNA levels in mouse plasma at multiple time points following surgery. LITT induced a modest increase in β-actin cfDNA at 1 h and 3 days post-procedure (*P* > .05 by 2-way ANOVA). (F) Quantification of *eGFP* circulating ctDNA normalized to β-actin cfDNA in mouse plasma at multiple time points post-surgery (*P* > .05 by 2-way ANOVA).

Since LITT increases local BBB permeability and allows systemic drugs to enter the brain,^24^ we asked whether this increase in BBB permeability might be bidirectional and potentially facilitate movement of molecules from the brain to the bloodstream, such as tumor material in the form of ctDNA. SB28 gliomas were implanted in the brains of B6 mice, and mice were subsequently treated with either LITT or sham surgery. Blood samples were collected at multiple time points to assess changes in ctDNA concentration, which was quantified using digital-droplet PCR (Figure 4A). We confirmed that LITT successfully ablated brain tumors by live bioluminescence imaging and observed that tumors in the LITT group were smaller than those in the sham group on day 6, with no significant differences observed on day −1 and day 13 (Figure 4B). Overall, tumors in both groups continued to grow throughout the blood collection period (Figure 4B). *eGFP*, a tumor-specific gene in the SB28 glioma line, was used as a ctDNA marker. *eGFP* ctDNA levels were significantly higher in the LITT group compared to the sham group as early as 1 h post-LITT and up to 14 days post-LITT (Figure 4C). We defined ctDNA positivity at >5 copies/µL due to assessed data showing reliable quantification at or above this threshold. Previous mouse xenograft studies have reported total ctDNA levels of approximately 8.4 copies/µL in mice bearing 1-2 g tumors.^25^ The positive detection rate was higher in the LITT group than in the sham group at 1 h after surgery (6/11 vs. 1/11, Figure 4D). LITT induced a modest increase in β-actin cfDNA at 1 h and 3 days post-LITT compared with sham surgery (Figure 4E). When eGFP ctDNA was normalized to β-actin cfDNA (Figure 4F), eGFP ctDNA remained elevated relative to the sham group at all 4 time points. Overall, these findings demonstrate that the LITT-triggered increase in BBB permeability improves ctDNA detection sensitivity in mice, supporting the hypothesis that such permeability changes are bidirectional, facilitating both molecular entry into and release from the brain.

## Discussion

The BBB hinders the flow of drugs between the blood and the brain parenchyma, limiting brain and brain tumor penetration by therapies. Therefore, numerous strategies have been designed to modulate the BBB and improve drug penetration.^26^ LITT has attracted attention given its potential for minimally invasive brain tumor ablation with non-inferior outcomes when compared with classical open surgery.^27–31^ Our group had previously demonstrated that LITT can increase BBB permeability for up to 4 weeks, which is mechanistically associated with reduced TJ integrity and enhanced transcytosis in brain endothelial cells.^9^ However, the detailed mechanistic basis, along with the spatial distribution and temporal persistence of these effects, had not yet been fully characterized.

Here, leveraging our previously established LITT murine model,^9^ our scRNA-seq analysis revealed that LITT induces transcriptional changes in brain endothelial cells characterized by downregulation of TJ-associated pathways and upregulation of pathways involved in caveolae-mediated transcytosis (Figure 1). These findings are consistent with our prior histological and ultrastructural observations that LITT disrupts junctional integrity and enhances vesicular transport, thereby promoting BBB permeability.^9^ Despite the change in TJ- and transcytosis-related pathways, substantial overlap in gene expression persisted between control and LITT-treated endothelial cells. This pattern likely reflects spatially restricted BBB opening that decreases with increasing distance from the laser ablation core, rather than uniform BBB disruption across the vasculature.

While previous studies have linked Wnt/β-catenin signaling to BBB stabilization,^22^^,^^32^^,^^33^ our finding of Wnt/β-catenin pathway upregulation after LITT may instead represent a compensatory attempt to restore junctional integrity. In addition, our transcriptomic analysis revealed enrichment of complement activation and neutrophil chemotaxis pathways, suggesting that endothelial responses to LITT extend beyond barrier regulation. These findings suggest that, beyond altering barrier integrity, LITT may also reshape the local immune environment, raising the possibility that endothelial responses contribute to enhanced immune activation following ablation.

Following LITT, claudin-5 expression in brain endothelial cells decreased sharply within the first week, with partial recovery by day 14 and complete restoration by day 21 (Figure 2). We also demonstrated that this effect was restricted to ∼100 µm around the ablation core. This transient and spatially confined TJ disruption highlights the temporal dynamics of BBB remodeling after thermal ablation. Nevertheless, Cldn5 does not show a significant transcriptional change in the single-cell RNA-seq data obtained one week after LITT. This discrepancy likely reflects spatial averaging across endothelial cells at varying distances from the lesion, temporal differences between transient transcriptional responses and more persistent protein-level changes, and technical differences between scRNA-seq and in situ histological analyses.

LITT increased caveolae-mediated transcytosis in brain endothelial cells, peaking at day 14 and returning to baseline by day 21, coinciding with downregulation of Mfsd2a (Figure 3). These results suggest that Mfsd2a contributes not only to baseline suppression of transcytosis but also to LITT-induced BBB permeability. Downregulation of Mfsd2a has been observed in other forms of CNS injury, including surgical brain injury and intracranial hemorrhage, suggesting that endothelial stress responses triggered by tissue injury may contribute to reduced Mfsd2a expression.^34^^,^^35^ In addition, upstream regulatory pathways such as Wnt/β-catenin signaling, which was among the top-enriched pathways in our transcriptomic analysis (Figure 1E), may further modulate Mfsd2a expression and transcytotic activity following LITT. Future studies aimed at validating the functional necessity of Mfsd2a in LITT-mediated endothelial transcytosis and dissecting the mechanisms linking injury, Wnt/β-catenin signaling, and Mfsd2a regulation will be important to fully understand BBB responses to LITT.

Using human GBM tissues, we found that endothelial caveolae density is overall very low in both normal brain and nonenhancing GBM regions, consistent with the well-documented suppression of baseline transcytosis in the healthy brain microvasculature.^23^ Notably, enhancing GBM regions showed a modest increase in caveolae density compared with both nonenhancing tumor regions and normal brain (Figure S2). While the observed regional heterogeneity reflects only a modest elevation in transcytosis activity, these descriptive findings contextualize the potential for therapeutic interventions, such as LITT, to further modulate BBB permeability by enhancing transcytosis, which requires further validation.

While this study focused on endothelial cell components of the BBB, other cell types in the tumor microenvironment might play a role in mediating LITT’s effect. Pericytes play a key role in mediating the BBB’s permeability and interact closely with endothelial cells. In fact, the endothelial expression of Mfsd2a has been shown to be modulated by endothelial-pericyte interactions.^7^ However, the impact of laser ablation on pericyte function in the surrounding areas of the ablation regions remains unknown. Further, neural activity has also been shown to modulate the BBB,^36^ with the BBB opening being observed in the setting of abnormally high neural activity in epilepsy.^37^ However, LITT’s effect on neurovascular coupling remains largely uncharacterized, and future studies should focus on further studying this relationship. The role of macropinocytosis—a less-studied, actin-dependent form of bulk endocytosis used by GBM cells for nutrient uptake—in response to LITT also remains unknown.^38^

There is great interest in deploying BBB opening technologies to enable drug delivery for molecules that otherwise would not readily cross the BBB.^39^ Our group has demonstrated this in the clinical setting with doxorubicin in recurrent GBM. Interestingly, in a Phase II trial, LITT followed by weekly low-dose doxorubicin initiated post-LITT demonstrated a significant overall survival benefit compared to historical controls treated with bevacizumab or LITT with standard salvage chemotherapy.^40^

Our findings reveal that LITT induces a bidirectional increase in BBB permeability, as evidenced by the enhanced release of tumor-derived ctDNA into the circulation (Figure 4). Plasma ctDNA levels in mice treated with LITT were significantly higher than those in sham controls, with detection occurring as early as 1 h and up to day 14, thereby overcoming the challenge of low ctDNA detectability in glioma models. Prior work with focused ultrasound has similarly shown that BBB opening enhances ctDNA detection.^41^ In contrast, LITT not only increases BBB permeability but also ablates tumor tissue. The peak in ctDNA at 3 days likely reflects rapid tumor cell death, which occurs earlier than maximal BBB permeability. LITT induced a relatively specific increase in tumor-specific cfDNA, likely because the primary target of LITT in these experiments was the brain tumor. Therefore, LITT may release a spatially broader repertoire of tumor-derived molecules than those that may typically be found in focal stereotactic biopsies and thus provide dual utility for both therapeutic and biomarker applications. As the first study to demonstrate LITT’s role in enhancing ctDNA detection, our results suggest that LITT could enable more comprehensive tumor mutation profiling by ablating multiple sites of tumors, though further human studies are needed to validate its clinical applicability for GBM management.

Our results have several limitations. We demonstrate that LITT can impair TJ integrity and increase transcytosis in a murine model. However, mice have different BBBs from humans, and the effect of LITT in human brains might be different. For scRNA-seq, immunofluorescence, and TEM experiments, sham controls involved only a burr hole. These controls did not capture potential effects of tissue disruption caused by probe insertion and should be taken into account when interpreting the findings. We also evaluated the presence, magnitude, and density of caveolae in human brains harboring GBMs. However, the variation of this process between primary and recurrent GBMs, as well as its role in different brain regions, remains uncharacterized. The analysis of LITT-treated human GBM samples is essential for further validation, but their acquisition is particularly challenging.

In summary, this study demonstrates that LITT can temporarily and reversibly increase BBB permeability by decreasing TJ integrity and increasing transcytosis with an effect lasting for up to 2 weeks and up to 100 µm away from the ablation zone in part due to Mfsd2a suppression. Further, this provides a window of opportunity that can be exploited to facilitate the potentially more spatially comprehensive detection of ctDNA and improve the effectiveness of adjuvant therapies being combined with LITT in future human clinical trials.

## Supplementary Material

noag080_Supplementary_Data

## Data Availability

scRNAseq data deposited to GEO (accession number GSE329855).
