# Supplementary material for: Laser interstitial thermal therapy enhances bidirectional blood-brain barrier permeability in glioblastoma
Source: Neuro Oncol. 2026 Apr 13;28(7):1649–61. doi: 10.1093/neuonc/noag080 (PMC13338339; doi:10.1093/neuonc/noag080)
Supplement: noag080_Supplementary_Data [file noag080_supplementary_data.zip › Supplementary Material 20260127 YF.docx]

**Supplementary Material**

**Supplementary Figure 1** Representative images of Evans Blue staining and GFP signal in SB28 tumors 7 days after LITT or sham treatment. Dashed lines demarcate the tumor.

**Supplementary Figure 2** Endothelial cell transcytosis in untreated human GBM specimens. (A) Schematic demonstrating sample collection sites at enhancing (blue) and non-enhancing (green) regions in human brain harboring GBM and representative transmission electron micrographs of caveolae in endothelial cells (E) facing the lumen (L) in normal brain and tumor regions. (B) Quantification of luminal caveolae density (mean +/- SEM) in endothelial cells in indicated regions. 10 capillary cross-sections quantified per sample. Scale bar: 100 nm.
