# Supplementary figures and images for: Laser interstitial thermal therapy enhances bidirectional blood-brain barrier permeability in glioblastoma
Source: Neuro Oncol. 2026 Apr 13;28(7):1649–61. doi: 10.1093/neuonc/noag080 (PMC13338339; doi:10.1093/neuonc/noag080)

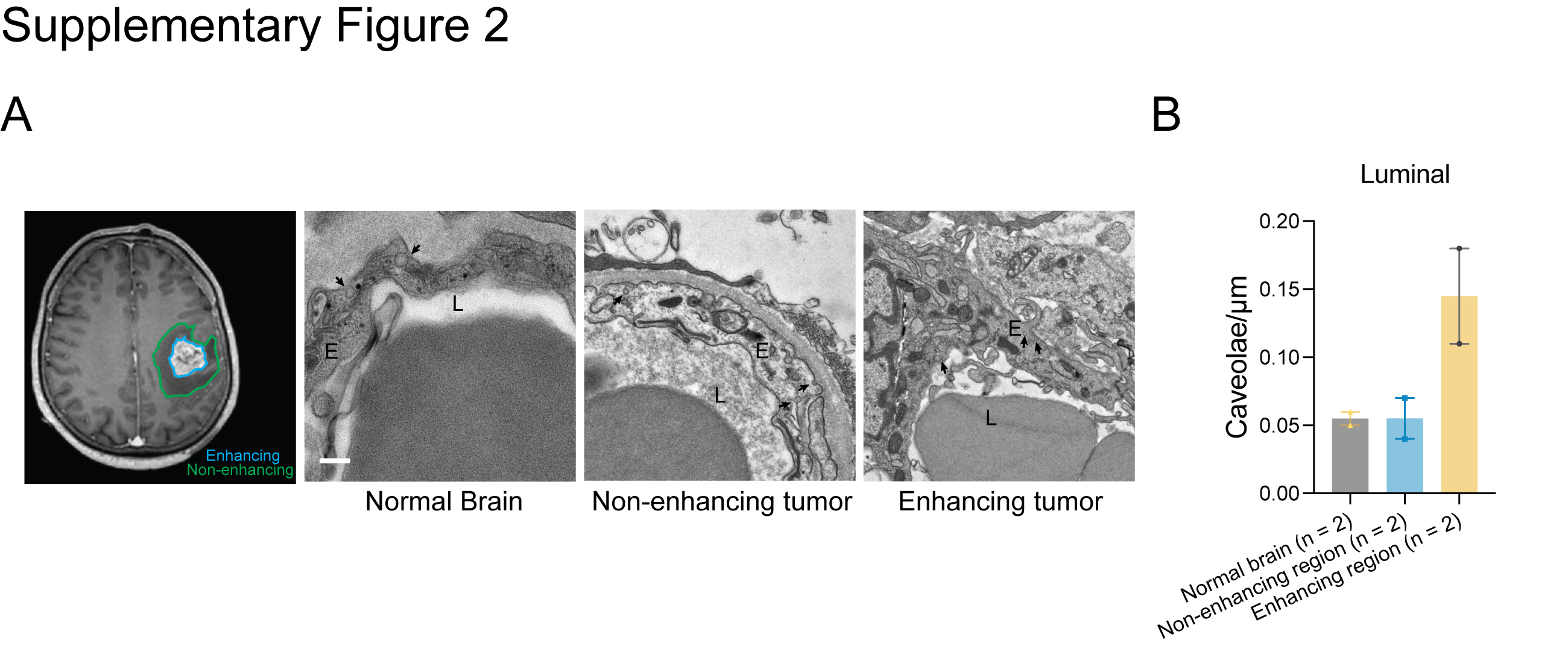

Supplement: noag080_Supplementary_Data [file noag080_supplementary_data.zip › supp fig human TEM.tif]

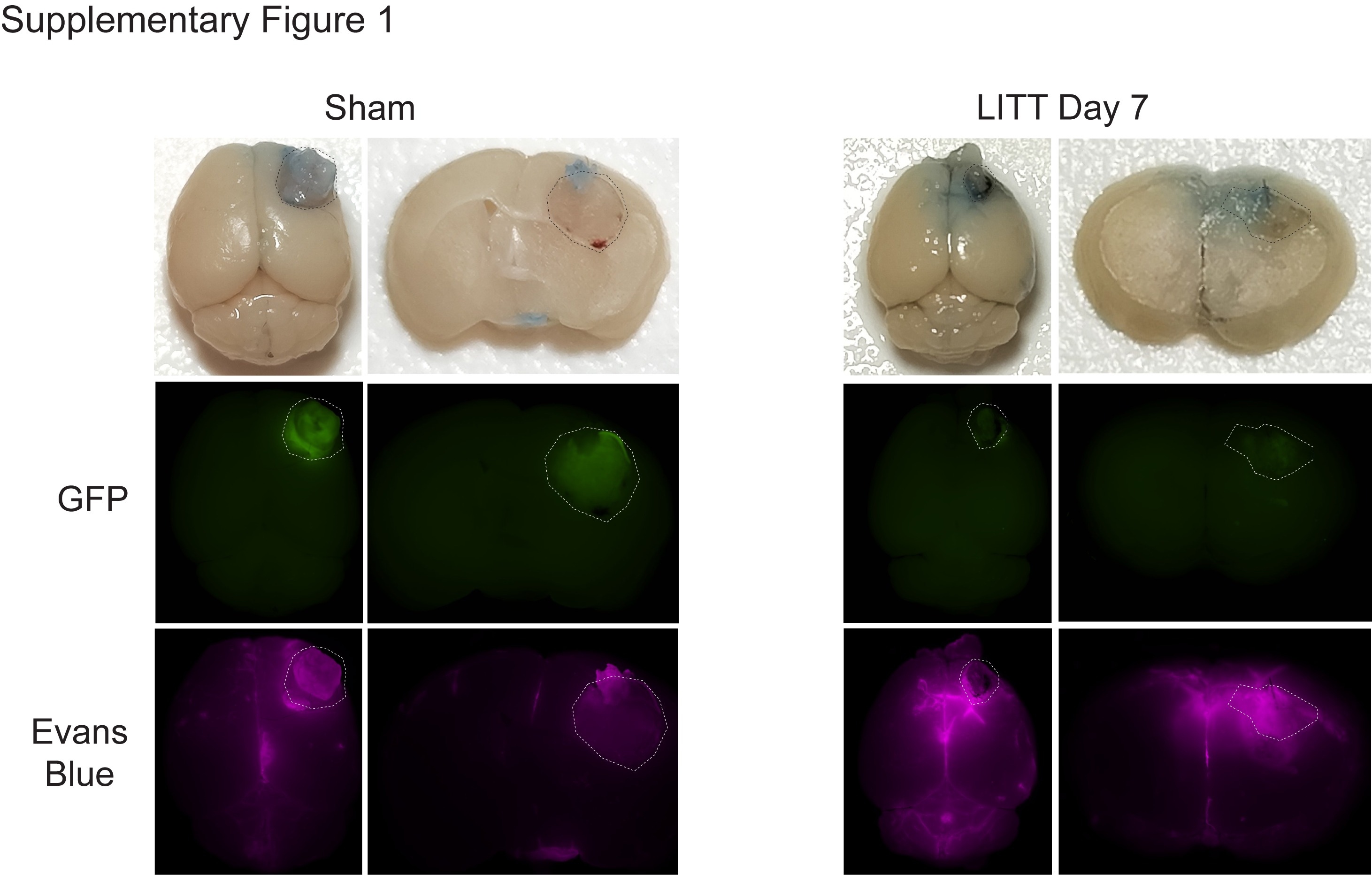

Supplement: noag080_Supplementary_Data [file noag080_supplementary_data.zip › supp_fig_evans_blue.tif]
